# Local Application of Semaphorin 3A Combined with Adipose-Derived Stem Cell Sheet and Anorganic Bovine Bone Granules Enhances Bone Regeneration in Type 2 Diabetes Mellitus Rats

**DOI:** 10.1155/2019/2506463

**Published:** 2019-07-31

**Authors:** Xiaoru Xu, Kaixiu Fang, Lifeng Wang, Xiangwei Liu, Yuchao Zhou, Yingliang Song

**Affiliations:** State Key Laboratory of Military Stomatology & National Clinical Research Center for Oral Diseases & Shaanxi Engineering Research Center for Dental Materials and Advanced Manufacture, Department of Implantology, School of Stomatology, The Fourth Military Medical University, Xi'an, 710032 Shaanxi, China

## Abstract

Bone tissue regeneration is considered to be the optimal solution for bone loss. However, diabetic patients have a greater risk of poor bone healing or bone grafting failure than nondiabetics. The purpose of this study was to investigate the influence of the complexes of an adipose-derived stem cell sheet (ASC sheet) and Bio-Oss® bone granules on bone healing in type 2 diabetes mellitus (T2DM) rats with the addition of semaphorin 3A (Sema3A). The rat ASC sheets showed stronger osteogenic ability than ASCs *in vitro*, as indicated by the extracellular matrix mineralization and the expression of osteogenesis-related genes at mRNA level. An ASC sheet combined with Bio-Oss® bone granules promoted bone formation in T2DM rats as indicated by microcomputed tomography (micro-CT) and histological analysis. In addition, Sema3A promoted the osteogenic differentiation of ASC sheets *in vitro* and local injection of Sema3A promoted T2DM rats' calvarial bone regeneration based on ASC sheet and Bio-Oss® bone granule complex treatment. In conclusion, the local injection of Sema3A and the complexes of ASC sheet and Bio-Oss® bone granules could promote osseous healing and are potentially useful to improve bone healing for T2DM patients.

## 1. Background/Introduction

Bone regeneration of bone defects is a challenge in patients with type 2 diabetes mellitus (T2DM). Diabetic patients have a greater risk of poor bone healing or bone grafting failure than nondiabetics [1–3]. Hundreds of millions of people suffer from diabetes, and China has the largest amount of diabetic patients in the world [4]; therefore, there is a high demand for improving the healing of alveolar bone defects in T2DM patients. In addition to traditional tissue transplants like autografts, allografts, and xenografts, stem cell-based tissue engineering of bone has become a brand-new and prospective remedy for bone healing. Many kinds of MSCs, such as bone marrow mesenchymal stem cells (BMSCs) [5], adipose-derived stem cells (ASCs) [6], human umbilical cord mesenchymal stromal cells [7], and human periodontal ligament stem cells (PDLSCs) [8] have been used to improve the bone healing in diabetics. ASCs have a good capacity for self-renewal, have a multipotential ability, are abundantly available, and are less likely to cause donor-associated morbidity [9–11]; thus, they provide promising seed cells for bone tissue engineering. Studies have proved that the local application of ASCs could enhance bone regeneration in the T2DM model [12, 13]. However, T2DM can affect biological characteristics and osteoblastic differentiation of MSCs through many factors [14, 15]. Measures should be taken to improve the osteogenic ability of ASCs in T2DM. Cell sheet engineering is one of the most promising approaches of tissue engineering in recent years. It can perfectly preserve cultured cells, extracellular matrix (ECM), and cell-cell and cell-ECM connections, avoiding the use of enzymes [16]. Semaphorin 3A (Sema3A) is a member of the semaphorin family. Researches show that Sema3A can promote osteogenic differentiation and inhibit osteoclast differentiation, in addition to the important role on neurological development and healing [17, 18]. Our previous study showed that overexpression of Sema3A in ASCs significantly enhanced the osteogenic ability of ASCs [19]. Bio-Oss® bone granules are anorganic bovine bone substitutes that are widely used in clinics due to their osteoconductivity and good biological compatibility [20, 21]. To compensate for their poor osteoinductivity, Bio-Oss® bone granules can be used as a scaffold in combination with MSCs. The present study assessed the osteogenic capacity of adipose-derived stem cell sheets (ASC sheets) *in vitro*. ASC sheets and Bio-Oss® bone granules were used to make tissue-engineered bone and were applied to T2DM rats. We found that ASC sheets with strong osteogenic capacity could promote bone healing in the T2DM model. Besides, the local injection of Sema3A could further improve bone regeneration in the T2DM model. Our study has revealed that tissue engineering of bone which was established using an ASC sheet, Bio-Oss® bone granules, as well as Sema3A holds a promising approach for bone regeneration in the future.

## 2. Materials and Methods

### 2.1. Animals

All animal experimental procedures were conducted in accordance with the committee guidelines of the Laboratory Animal Care & Welfare Committee, School of Stomatology, Fourth Military Medical University, China. Four-week-old Sprague-Dawley rats were used for the isolation of ASCs. Eight-week-old male SD rats were purchased to induce T2DM models and then used in animal experiments. Animals were maintained in specific pathogen-free conditions under a 12 h light/dark cycle with access to a high-fat diet, at 26°C and a humidity of 30-70% throughout the study.

### 2.2. Isolation and Characterization of Adipose-Derived Stem Cells (ASCs)

After being executed by cervical dissection, the rats were submerged in 70% ethanol for 5 min. The inguinal fat pads were obtained under sterile conditions. After being washed with phosphate-buffered saline (PBS) (Gibco, USA), the fresh adipose tissue was minced into paste, digested in an equal volume of 0.1% collagenase type I (Sigma-Aldrich, USA) at 37°C for 40 min, and filtered with a sterile stainless steel sieve (75 *μ*m mesh). The filtrate was centrifuged at 1,200 rpm for 5 min, resuspended in 10 mL PBS, and centrifuged again. The cells were cultured in a complete medium consisting of *α*-minimum essential media (*α*-MEM) (Gibco, USA), 10% fetal bovine serum (Sijiqing, China), and 1% penicillin/streptomycin (HyClone, USA) and incubated at 37°C in a humidified atmosphere of 5% CO_2_ and 95% air. Cells of passage 3 were used for the follow-up experiments.

To determine the multilineage differentiation capacity of the ASCs, the cells were plated in six-well culture plates and the culture medium was changed to osteogenic or adipogenic medium when the cells reached 80% confluence. The osteoinductive medium was prepared using *α*-MEM (Gibco, USA) supplemented with 10% FBS (Sijiqing, China), 0.1 mM dexamethasone (Sigma-Aldrich, USA), 5 mM *β*-glycerophosphate (Sigma-Aldrich, USA), 50 *μ*g/mL L-ascorbic acid (Sigma-Aldrich, USA), and 1% penicillin/streptomycin (HyClone, USA). The adipogenic medium was composed of *α*-MEM (Gibco, USA) containing 10% FBS (Sijiqing, China), 1% penicillin/streptomycin (HyClone, USA), 0.5 mM 3-isobutyl-1-methylxanthine (IBMX, France), 1 *μ*M dexamethasone (Sigma-Aldrich, USA), 0.1 mM indomethacin (Sigma-Aldrich, USA), and 10 *μ*g/mL insulin (Sigma-Aldrich, USA). The osteogenic or adipogenic induction medium was changed every 3 days. The calcium deposits yielded by the ASCs were visualized by Alizarin Red staining (Sigma-Aldrich, USA) after osteogenic induction for 28 days, while lipid droplets were revealed by Oil Red O staining (Sigma-Aldrich, USA) after adipogenic induction for 14 days.

### 2.3. Immunophenotype of ASCs

Some 1 × 10^6^ third-passage ASCs were fixed with 4% paraformaldehyde for 15 min and then incubated with phycoerythrin- (PE-) or fluorescein isothiocyanate- (FITC-) conjugated monoclonal antibodies for rat CD34 (R&D Systems, USA), CD44 (Santa Cruz Biotechnology, USA), CD45 (eBioscience, USA), and CD90 (eBioscience, USA) at room temperature for 1 h and then at 4°C in the dark. The labeled ASCs were assessed using a flow cytometer (Beckman Coulter, USA). The monoclonal antibodies CD44-PE and CD90-FITC were used to identify the mesenchymal phenotype, and CD34-PE and CD45-PE were applied to exclude the hematopoietic and angiogenic lineages.

### 2.4. Fabricating ASC Sheets

The third-generation ASCs were seeded at 1 × 10^6^ cells/well in 6-well plates. After reaching about 90% confluence, the basal medium was changed to a cell sheet induction medium, which was composed of *α*-MEM (Gibco, USA), 10% bovine fetal serum (Sijiqing, China), 1% penicillin/streptomycin (HyClone, USA), and 50 mg/mL vitamin C (Kehao, China). ASCs were cultured for 7 to 10 days, and the nutrient solution was replaced every 2 to 3 days. When the curly edge appeared at the plate rim, the whole cell sheets were peeled off with a scraper or tweezers. The ASC sheets were always kept moist during the peeling process.

### 2.5. Osteogenesis Capability of ASCs and ASC Sheets

For *in vitro* osteogenic differentiation analysis, both the ASC group and the ASC sheet group were started with a 1 × 10^6^ cells/well seeding in 6-well plates. The ASC group was osteoinducted once the cell confluence reached 90%, while the ASC sheet group was osteoinduced only after a 7-day cell sheet induction. The osteoinductive medium was prepared using *α*-MEM (Gibco, USA) supplemented with 10% FBS (Sijiqing, China), 0.1 mM dexamethasone (Sigma-Aldrich, USA), 5 mM *β*-glycerophosphate (Sigma-Aldrich, USA), 50 *μ*g/mL L-ascorbic acid (Sigma-Aldrich, USA), and 1% penicillin/streptomycin (HyClone, USA).

#### 2.5.1. Osteogenesis Staining

Both groups were subjected to ALP staining (Leagene, China) at the 7th day of osteogenic induction and to Alizarin Red staining (Sigma-Aldrich, USA) at the 28th day. The results were observed and recorded by digital camera (Nikon, Japan).

#### 2.5.2. Real-Time RT-qPCR

At the 7th day of osteogenic differentiation, the relative mRNA expressions of alkaline phosphatase (ALP), bone morphogenetic protein 2 (BMP2), osteocalcin (OCN), and runt-related transcription factor 2 (Runx-2) in the ASC group and in the ASC sheet group were determined. The total RNA of ASCs and ASC sheets was extracted using the TRIzol Reagent (Invitrogen, USA) according to the manufacturer's protocol. After quantification by optical density measurement, 1 *μ*g total RNA was converted to cDNA using the PrimeScript™ RT Reagent Kit (Takara, Japan). RT-PCR was performed using the SYBR Premix Ex Taq™ II Kit (Takara, Japan) in a quantitative PCR system (Bio-Rad, USA) under the following conditions: 3 min of denaturation at 95°C, 40 rounds of 10 s of annealing at 95°C, and 30 s of extension at 60°C. The primers used in the present study are listed in Table 1; GAPDH was monitored as a housekeeping gene. The results were evaluated by the CFX96™ RT-PCR System (Bio-Rad, USA).

### 2.6. In Vitro Osteogenesis of ASC Sheets with Sema3A

The procedures for ASC isolation and ASC sheet fabrication were the same as mentioned above. After a 7-day cell sheet induction, ASC sheets were treated with osteoinductive medium as mentioned above with or without 1 *μ*g/mL Sema3A (PeproTech, USA). The treatment groups were then named the control group and the Sema3A group. ALP staining and Alizarin Red staining, as well as osteogenesis-related gene expression were tested as mentioned above.

### 2.7. Induction of T2DM Rat Model

A high-fat diet with 69.5% basal feed, 10.0% sucrose, 10.0% egg yolk granules, 0.5% cholesterol, and 10.0% lard (Experimental Animal Center of the Fourth Military Medical University) for four weeks and a single low dose (30 mg/kg) of streptozotocin (STZ) via intraperitoneal injection were administered to rats to induce type 2 DM models as previously described [22]. After 7 days of STZ injection, blood was collected by tail cutting to test the random plasma glucose levels (PGLs) using a glucometer. Rats with PGL above 16.7 mmol/L were considered as diabetics, but PGL below this value were excluded from the experiment.

### 2.8. Characteristics and Preparation of Implants

#### 2.8.1. ASC+Bone Granule Complex

**Figure 1:**
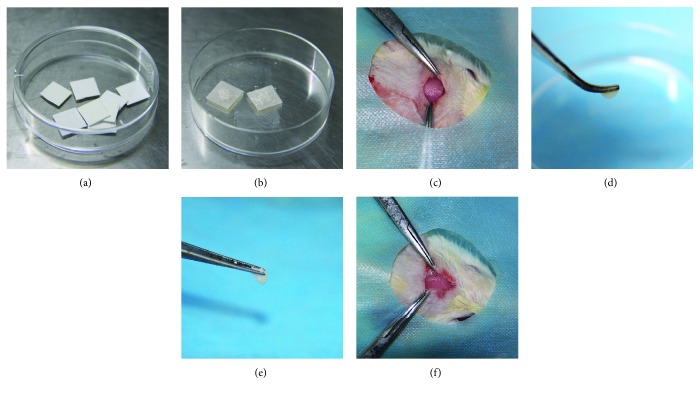
Preparation and transplantation of different implants. (a–c) ASC+bone granule complex: (a) 7 mm × 7 mm biomembranes, (b) 3 × 10^6^ ASCs were seeded on 0.02 g bone granules above the biomembrane, and (c) the ASC+bone granule complex together with the biomembrane were transplanted in the CSD of T2DM rat. (d–f) ASC sheet+bone granule complex: (d) ASC sheet pellet which started from 3 × 10^6^ ASCs in 60 mm petri dishes and cultured in cell sheet induction medium for 7 days, (e) the complex of the ASC sheet and 0.02 g bone granules, and (f) the ASC sheet+bone granule complex was transplanted in CSD of the T2DM rat and covered by the 7 mm × 7 mm biomembrane.

Biomembranes (Heal-All, China) were cut into 7 mm × 7 mm squares. Bio-Oss® bone granules (0.02 g) (Geistlich, Switzerland) were loaded on the biomembrane. 3 × 10^6^ ASCs were dropped on bone granules and cocultured for 4 hours (Figures 1(a)–1(c)).

#### 2.8.2. ASC Sheet+Bone Granule Complex

3 × 10^6^ ASCs were seeded into 60 mm petri dishes and induced to the ASC sheet as mentioned earlier for 7 days. Then, 0.02 g bone granules (Geistlich, Switzerland) were mixed evenly with each ASC sheet in a 1.5 mL EP tube (Figures 1(d)–1(f)).

#### 2.8.3. Scanning Electron Microscopy (SEM) Observation of the Two Complexes

The surface morphologies of the two kinds of complexes were observed by JEOL JSM-6700F Field Emission SEM (JEOL Ltd., Japan).

### 2.9. Implantation of Two Complexes in T2DM Rats

A total of 20 rats with T2DM were randomly divided into two groups (*n* = 10): the ASC+bone granule group and the ASC sheet+bone granule group. Animals were anesthetized by an intraperitoneal injection of 2% pentobarbital sodium solution (Sigma-Aldrich, USA) (0.25 mL/100 g body weight). Following shaving and sterilization, a 5 mm critical-sized calvarial defect (CSD) was drilled carefully penetrating through the calvarial bone without damage to the dura mater. CSDs were randomly filled with the different complexes in the two groups. The complexes were placed over the dura mater and covered with the 7 mm × 7 mm biomembrane (Heal-All, China). The periosteum and skin were sutured separately with 4-0 silk sutures. Antibiotics based on body weight were administered for 3 consecutive days post surgery. The healing process was 4 or 8 weeks, then the rats were euthanized with an overdose of anesthetic. Calvarial specimens were harvested, fixed in 4% paraformaldehyde for 2 days, and analyzed by microcomputed tomography (micro-CT) and histomorphology.

### 2.10. Micro-CT Scanning

**Figure 2:**
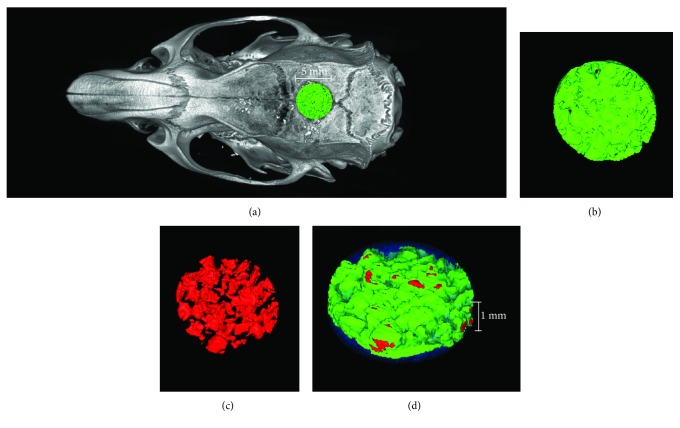
Definition of the region of interest (ROI). (a) The ROI was defined as a cylinder with a radius of 5 mm and a height of 1 mm from the surgery area. (b) Tissue with a CT value between 700 and 2000 Hu was defined as new bone; green=new bone. (c) Tissue with a CT value above 2000 Hu was defined as Bio-Oss® bone granules; red=bone granules. (d) Three-dimensional reconstruction of the ROI, translucent blue=CT value below 700 Hu.

A micro-CT scanner (Inveon CT, Siemens, Germany) was used to scan the samples at a scanning resolution of 56 *μ*m to evaluate bone formation in the CSDs of the T2DM rats. Three-dimensional models were reconstructed from the micro-CT scanning datasets for the quantitative analysis of bone formation within the CSDs (Figures 2(a) and 2(d)). The region of interest (ROI) was defined as a cylinder with a radius of 5 mm and a height of 1 mm (about the full thickness of the calvarial bone) from the surgery area. The Inveon Research Workplace software package, version 2.2.0 (Siemens Healthcare GmbH, Erlangen, Germany) was used for 3D reconstruction of the image and data analysis. Tissue with a CT value between 700 and 2000 Hounsfield units (Hu) is defined as new bone (Figure 2(b)). Tissue with a CT value above 2000 Hu is defined as Bio-Oss® bone granules (Figure 2(c)). The bone volume/total volume (BV/TV), trabecular thickness (Tb.Th), trabecular number (Tb.N), and trabecular spacing (Tb.Sp) were calculated.

### 2.11. Histomorphologic Analyses

The calvarial specimens were decalcified in 17% EDTA in a 37°C incubator for 20-30 days until the bone tissue became soft and could be easily penetrated by needles. The EDTA was changed twice weekly. After embedding in paraffin, representative coronal sections were taken and hematoxylin and eosin (HE) staining was performed. The sections were observed using a stereo microscope (Olympus Corporation, Tokyo, Japan).

#### 2.11.1. Vascular Counting

Three HE-stained sections were selected from each sample. Three fields were randomly selected for each section and the number of blood vessels was counted at 40 times magnification. An average value was calculated.

### 2.12. Bone Healing in T2DM Rats with Sema3A

A total of 20 rats with T2DM were randomly divided into two groups (*n* = 10): the control group and the Sema3A group. CSDs were drilled penetrating through the calvarial bone and filled with the ASC sheet+bone granule complex as mentioned above in all the rats. Rats received a local injection of Sema3A (100 *μ*g/mL in sterile saline, 20 *μ*g/kg) into the surgery site in the Sema3A group or vehicle (sterile saline) in the control group on the 1st, 4th, and 7th day after operation. All animals were euthanized 4 or 8 weeks later with an overdose of anesthetic. Samples were harvested and examined by micro-CT and histomorphology. The procedure was the same as mentioned above.

### 2.13. Statistical Analysis

All experiments were repeated at least three times and the results were displayed as mean ± standard deviation. Comparisons were performed by Student's *t*-test or one-way ANOVA followed by LSD-*t*-test or Games-Howell test using SPSS 19.0 (SPSS Inc., USA). Significance was considered as *P* value < 0.05.

## 3. Results

### 3.1. Characterization of ASCs

**Figure 3:**
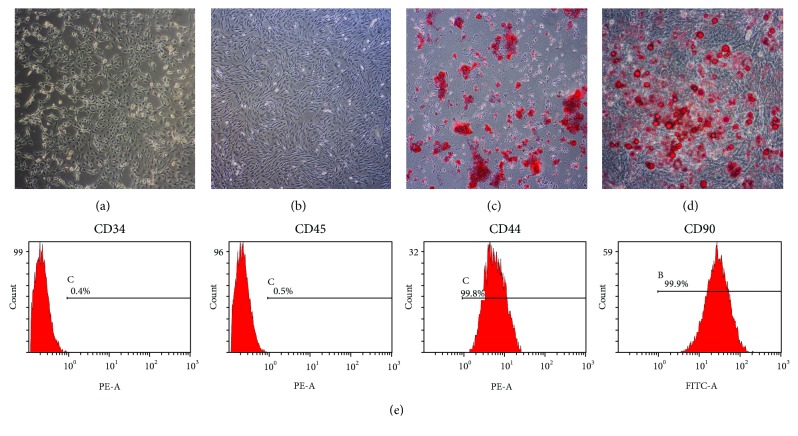
Characterization of ASCs. (a) Primary culture of ASCs (original magnification ×100). (b) Subculture of ASCs (P3, original magnification ×100). (c) Mineral node stained with Alizarin Red S (original magnification ×40). (d) Fat droplets stained with Oil Red O (original magnification ×200). (e) Flow cytometry analysis of ASC surface markers.

Primary culture of ASCs emerged as colonies with spindle-shaped morphology (Figure 3(a)). Cell population appeared to be more homogeneous by the third passage (P3, Figure 3(b)). In osteogenic culture, calcium nodules were stained with Alizarin Red S (Figure 3(c)). In adipogenic culture, intercellular lipid vacuoles were stained with Oil Red O (Figure 3(d)). ASCs were positive for the MSC markers CD44 (99.8% ± 0.1%) and CD90 (99.9% ± 0.1%) but negative for the hematopoietic or angiogenic markers CD34 (0.4% ± 0.2%) and CD45 (0.5% ± 0.2%) (Figure 3(e)).

### 3.2. Osteogenic Differentiation of the ASCs and ASC Sheets

**Figure 4:**
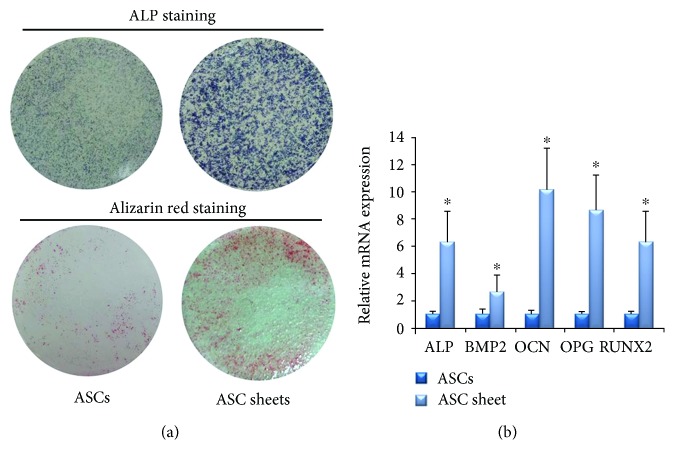
Osteogenic differentiation of the ASCs and ASC sheets. (a) ALP staining after osteogenic induction for 7 days and Alizarin Red staining after osteogenic induction for 28 days. (b) Osteogenesis-related gene expression quantified by RT-PCR after osteogenic induction for 7 days. Mean ± SD, *n* = 3, and ^∗^*P* < 0.05.

The results of ALP staining showed that ASC sheets after osteogenic induction for 7 days were deeper colored than ASCs (Figure 4(a)). The areas of mineralization nodules in the ECM of ASC sheets after osteogenic induction for 28 days were significantly larger and denser than ASCs (Figure 4(a)).

At the 7th day of osteogenic differentiation, the relative mRNA expressions of ALP, BMP2, OCN, OPG, and Runx2 in ASC sheets were higher than those in the ASC group (Figure 4(b)). The data of the two groups were statistically significant (*P* < 0.05).

### 3.3. In Vitro Osteogenesis of ASC Sheets with Sema3A

**Figure 5:**
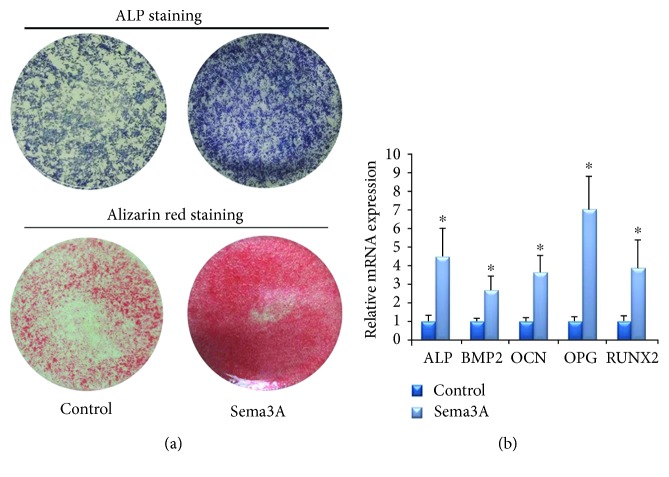
The effect of Sema3A on osteogenic differentiation of ASC sheets. (a) ALP staining after osteogenic induction for 7 days and Alizarin Red staining after osteogenic induction for 28 days. (b) Osteogenesis-related gene expression quantified by RT-PCR after osteogenic induction for 7 days. Mean ± SD, *n* = 3, and ^∗^*P* < 0.05.

To evaluate the effect of Sema3A on osteogenic differentiation, ASC sheets were treated with osteoinductive medium with Sema3A. Both ALP activity and deposition of calcified extracellular matrix were increased, as detected by ALP staining and Alizarin Red staining results (Figure 5(a)). In addition, the mRNA levels of osteogenic markers, including ALP, BMP2, OCN, OPG, and Runx2 were significantly higher in the Sema3A group after 7-day osteogenic inductions (Figure 5(b)). All these results verified that Sema3A significantly increased the osteogenic capacity of ASC sheets *in vitro*.

### 3.4. SEM Observation of Two Kinds of Complexes

**Figure 6:**
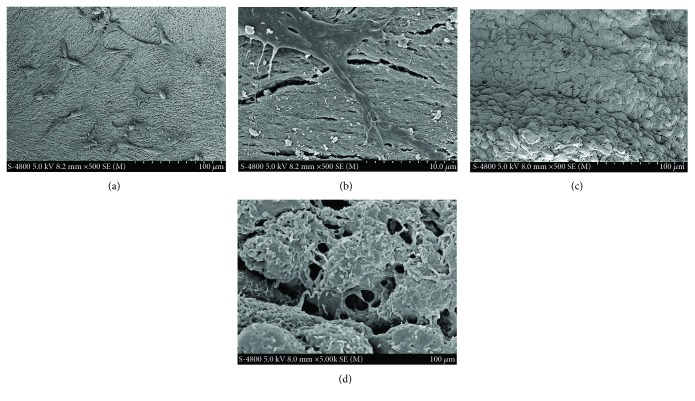
Surface morphologies of two kinds of complexes by SEM. (a, b) ASC+bone granule coculture complex. (c, d) ASC sheet+bone granule complex. Scale bar of (a) and (c), 100 *μ*m; scale bar of (b) and (d), 10.0 *μ*m.

In the ASC+bone granule complex, ASCs adhered tightly to the Bio-Oss® bone granules by protruding their projections on the surface of the bone granules (Figures 6(a) and 6(b)). In the ASC sheet+bone granule complex, numerous ASCs were densely populated in the ASC sheet and abundant cellular junctions were observed between the cells (Figures 6(c) and 6(d)). Under the same magnification, the ASC sheet+bone granule complex contained more cells.

### 3.5. Osseointegration of Different Types of Tissue Engineering Bone in T2DM Rats

#### 3.5.1. The ASC Sheet+Bone Granule Group and the ASC+Bone Granule Group

*(1) Rat Physical Health*. All the rats were successfully modeled. The average blood glucose of T2DM rats was 24.9 ± 2.8 mmol/L, and the average body weight was 375 ± 26.5 g before surgery. Blood glucose was stable throughout the experiment. All animals survived, and none showed signs of infection during the experiment.

**Figure 7:**
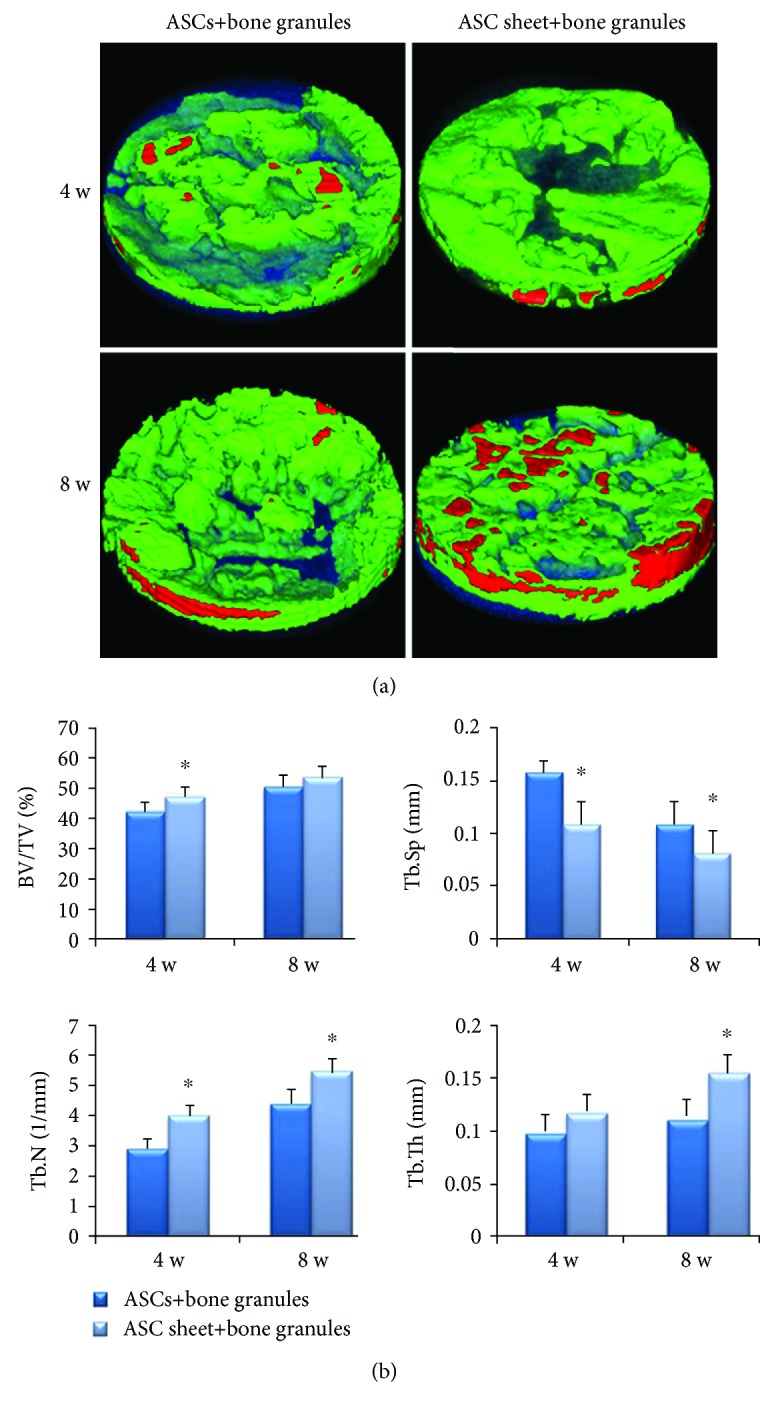
Micro-CT evaluation of the ASC+bone granule group and the ASC sheet+bone granule group. (a) Three-dimensional images of micro-CT reconstruction of ROI (a cylinder with a radius of 5 mm and a height of 1 mm). Green=new bone (CT value between 700 and 2000 Hu), red=bone granules (CT value above 2000 Hu), and translucent blue=trabecular spacing (CT value below 700 Hu). (b) BV/TV, Tb.Sp, Tb.N, and Tb.Th evaluation of ROI. One-way ANOVA, followed by LSD-*t*-test or the Games-Howell test; ^∗^*P* < 0.05. BV/BT, bone volume/total volume; Tb.Th, trabecular thickness; Tb.N, trabecular number; Tb.Sp, trabecular spacing; ROI, region of interest.

*(2) Micro-CT Analysis*. Three-dimensional images on micro-CT showed massive newly formed bone in both two groups (Figure 7(a)). More new bone was observed in the ASC sheet+bone granule group than in the ASC+bone granule group. All the BV/TV, Tb.Sp, Tb.N, and Tb.Th were statistically different (*P* < 0.05) between the two groups except BV/TV at 8 weeks and Tb.Th at 4 weeks (Figure 7(b)). The results showed that new bone formation was significantly improved in the ASC sheet+bone granule group.

**Figure 8:**
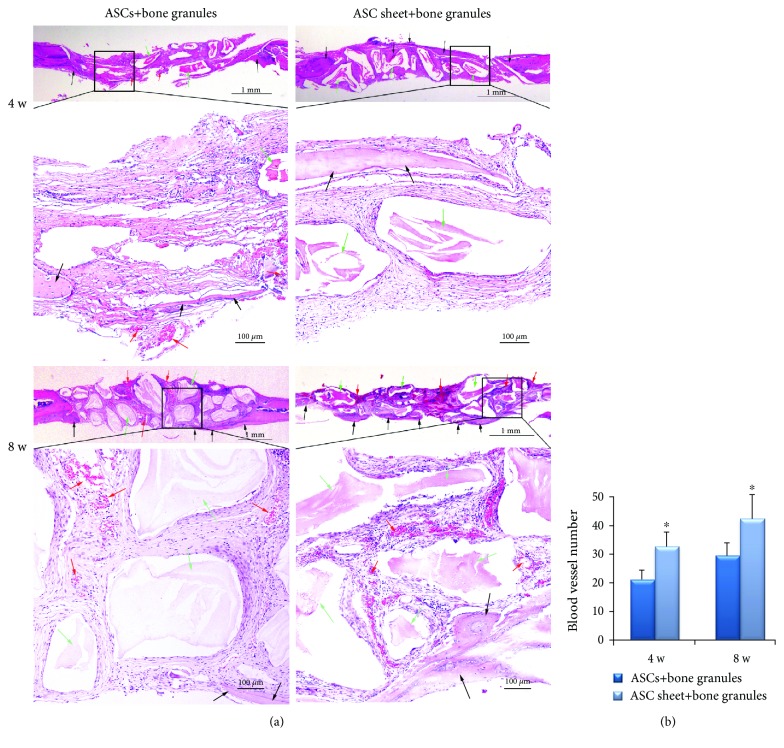
Histomorphologic analyses of the ASC+bone granule group and the ASC sheet+bone granule group. (a) New bone formation of the calvarial defect sections of the two groups was detected by H&E staining. Scale bar of upper images, 1 mm; scale bar of lower images, 0.5 mm. (b) Blood vessel number of the calvarial defect sections of the two groups. Black arrows indicate new bone, red arrows indicate blood vessels, and green arrows indicate bone granules; the lower panels are the magnifications of the insets in each group. Mean ± SD, *n* = 3, and ^∗^*P* < 0.05.

*(3) Histologic Analysis of New Bone within the CSDs*. To further investigate the newly formed bone within the CSDs, histologic analysis was performed using HE staining under light microscopy (Figure 8(a)). A large number of active osteoblasts and woven bone were observed around bone granules in both groups. New bone was observed only around the margin of CSDs in the ASC+bone granule group, while new bone started to grow into the central area in the ASC sheet+bone granule group at 4 weeks. At 8 weeks, the difference was more obvious with bone remodeling. In the ASC sheet+bone granule group, more mature new bone was creeping from the periphery to the center of the CSDs, and osseous islands and bridges were observed in the center of the CSDs. In addition, more blood vessels were observed in the ASC sheet+bone granule group than in the ASC+bone granule group (Figure 8(b)).

#### 3.5.2. The Sema3A Group and the Control Group

*(1) Rat Physical Health*. All the rats were successfully modeled. The average blood glucose of T2DM rats was 25.7 ± 3.4 mmol/L, and the average body weight was 383 ± 28.7 g before surgery. Blood glucose was stable throughout the experiment. All animals survived, and none showed signs of infection during the experiment.

**Figure 9:**
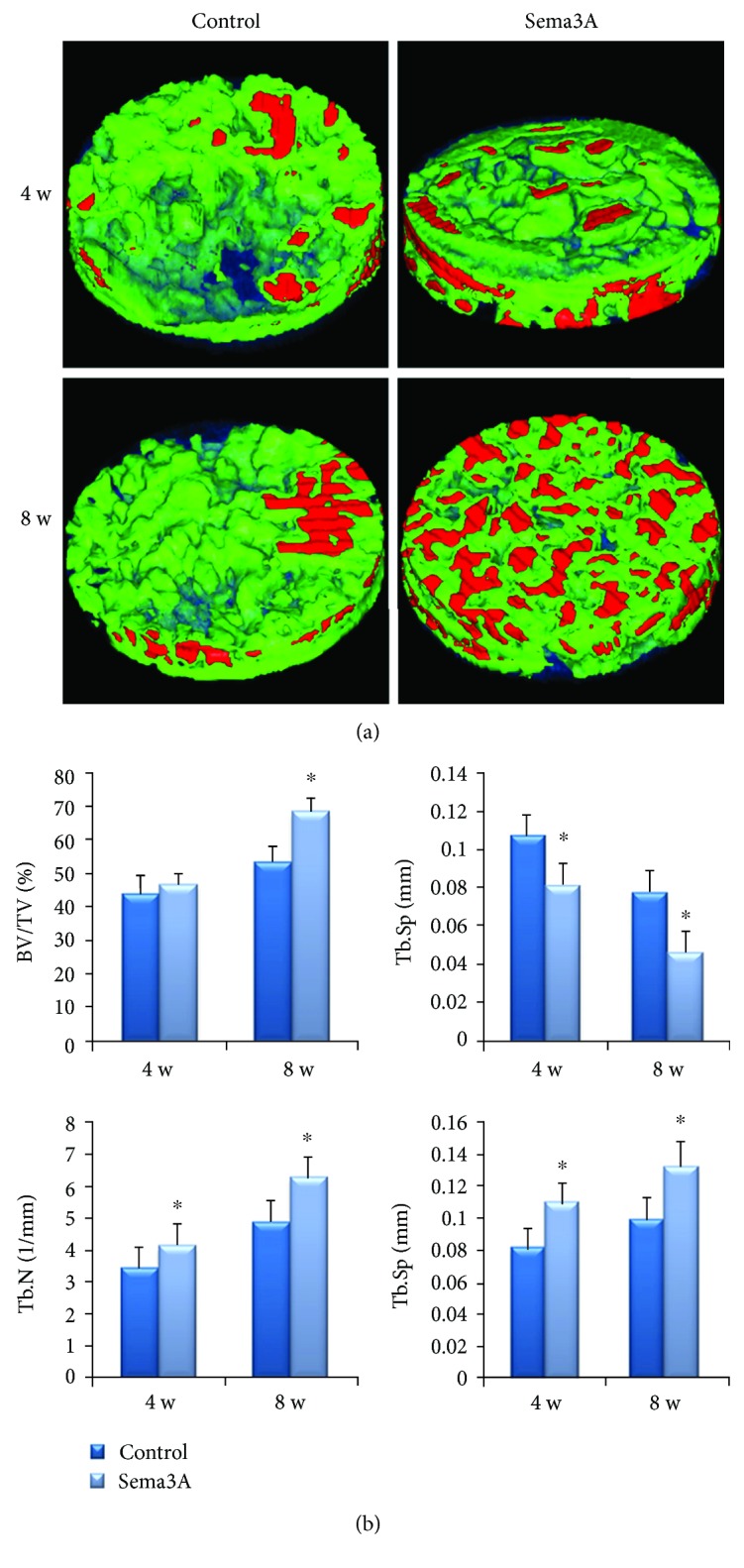
Micro-CT evaluation of the control group and the Sema3A group. (a) Three-dimensional images of micro-CT reconstruction of ROI (a cylinder with a radius of 5 mm and a height of 1 mm). Green=new bone (CT value between 700 and 2000 Hu), red=bone granules (CT value above 2000 Hu), and translucent blue=trabecular spacing (CT value below 700 Hu). (b) BV/TV, Tb.Sp, Tb.N, and Tb.Th evaluation of ROI. One-way ANOVA, followed by LSD-*t*-test or the Games-Howell test; ^∗^*P* < 0.05. BV/BT, bone volume/total volume; Tb.Th, trabecular thickness; Tb.N, trabecular number; Tb.Sp, trabecular spacing; ROI, region of interest.

*(2) Micro-CT Analysis*. Three-dimensional images on micro-CT showed massive newly formed bone in both two groups (Figure 9(a)). More new bone was observed in the Sema3A group than in the control group. At 8 weeks, the morphology of the bone granules was blurred and the new bone almost completely filled the bone defect area in the Sema3A group. Besides, more red area in the Sema3A group suggested that the degree of new bone mineralization was higher as well. The BV/TV, Tb.N, and Tb.Th were higher and Tb.Sp was less in the Sema3A group (Figure 9(b)). The results showed that Sema3A significantly improved new bone formation in the T2DM model.

**Figure 10:**
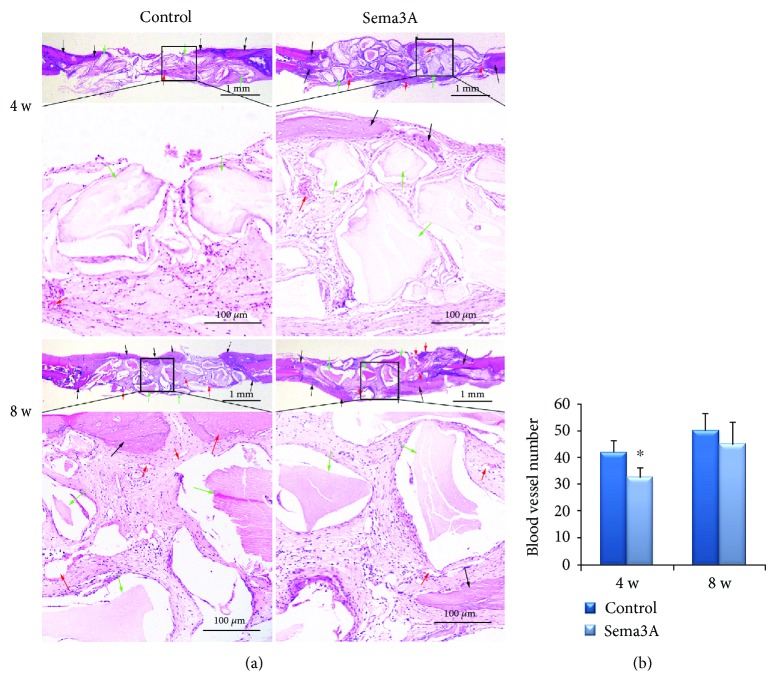
Histomorphologic analyses of the control group and the Sema3A group. (a) New bone formation of the calvarial defect sections of the two groups was detected by H&E staining. Scale bar of upper images, 1 mm; scale bar of lower images, 0.5 mm. (b) Blood vessel number of the calvarial defect sections of the two groups. Black arrows indicate new bone, red arrows indicate blood vessels, and green arrows indicate bone granules; the lower panels are the magnifications of the insets in each group. Mean ± SD, *n* = 3, and ^∗^*P* < 0.05.

*(3) Histologic Analysis of New Bone within the CSDs*. At 4 weeks, osseous islands were observed in the center of the CSDs in the Sema3A group, while new bone was observed only around the margin of CSDs in the control group. At 8 weeks, new bone in the Sema3A group was thicker and fused continuously, almost completely covering the bone defect area (Figure 10(a)). Less blood vessels were observed in the Sema3A group at 4 weeks while the difference was not significant at 8 weeks (Figure 10(b)).

## 4. Discussion

Quite a few T2DM patients suffer from impaired bone healing [1] and bone grafting failure [2], which is often associated with the suppression of osteogenic differentiation of MSCs [14] and thus become a crucial issue hindering clinical application of MSCs in patients with T2DM. In this study, we found that ASC sheets preserved more cells and had better osteogenic ability than ASCs *in vitro*. Importantly, based on critical calvarial defect repair in the T2DM model, we certified that ASC sheets improved bone regeneration *in vivo*. We further confirmed that Sema3A significantly increased the osteogenic capacity of the ASC sheets *in vitro* and *in vivo*. Taken together, our study highlights the promising effect of bone tissue engineering based on ASC sheets, Bio-Oss® bone granules, and Sema3A on bone healing in the T2DM model.

In bone tissue engineering, the traditional method of seeding MSCs onto scaffolds often results in a great loss of cells. In order to solve the problem, we loaded scaffolds on a biomembrane when seeding stem cells in a previous study [13], where cells that failed to attach to scaffolds could adhere to the biomembrane below, maximizing the utilization of stem cells *in vivo*. However, the effect is still limited and the prepared complexes with the specific biomembrane can hardly be adjusted to the irregular shape of bone defects in clinics. Cell sheet technology is an alternative approach of tissue engineering that binds cells tightly in a sheet form via temperature-responsive culture [23], electron beam irradiation [24], mechanical methods [25], or vitamin C [26] application, to prevent cell loss, provide an ideal microenvironment, and obtain a certain degree of mechanical strength for the seed cells by preserving both cell surface proteins and ECM to the utmost [27]. Therefore, we used cell sheet technology in this study.

Considering the low price, convenient operation, and satisfactory film-inductive effect, we constructed ASC sheets with vitamin C in this study. In the osteogenic experiments, ASC sheets showed better osteogenic ability with enhanced ALP activity, more calcium deposition, and the elevated expression level of osteogenesis-related genes. These results claimed a positive effect of cell sheets on the osteogenesis of ASCs, just as what has been proven with many other MSCs including BMSCs and PDLSCs [16, 28].

Stem cell sheets are now generally used as a periosteum, wrapping scaffolds to repair bone defects [29–34]. However, cells cannot be evenly distributed on the scaffolds in this way, and this method has limits on osteoinductivity. In this study, we thoroughly mixed ASC sheets and Bio-Oss® bone granules in an EP tube to make sure that ASCs exist in the center of the defect and intact ASC sheets act as a whole to contact the bone tissue of the host. The results of SEM showed that the ASC sheet+bone granule complex perfectly guaranteed the number of ASCs, intercellular connection, and cell-ECM connection. This may facilitate cell signaling, thereby promoting cell differentiation and new bone formation. Besides, the complexes can fit bone defects of different sizes and shapes in clinics only by adjusting the amount of the bone granules and cell sheets during the operation. Our study provided a novel strategy with high efficiency and convenience to perform bone tissue engineering only by mixing cell sheets and scaffold granules during surgery, which is very practical when applied in clinics.

Many *in vitro* and *in vivo* studies have shown that Sema3A can promote osteogenic differentiation and new bone formation [35–38]. Sema3A has a good curative effect on the osteoporosis model, the cortical bone defect model [17], and the rat osteoporotic fracture model [39], which can promote bone regeneration, increase bone mass, and reduce bone loss in injured parts. The mechanism may be that Sema3A binds to the neuropilin-1 (Nrp1) receptor and activates *β*-catenin, which promotes osteogenesis through the Wnt/*β*-catenin signaling pathway [17]. In addition, Hayashi et al. confirmed that Sema3A, like OPG, also has an inhibitory effect on osteoclast formation with OPG-deficient mice; this may be due to the fact that the binding of Sema3A to Nrp1 inhibited osteoclast differentiation by intervening in the ITAM and RhoA pathway [17]. Consistent with a previous study, our group showed that the stimuli of Sema3A can promote the osteogenic ability of the ASC sheets of 7-day induction as revealed by improved ECM mineralization and a higher expression of osteogenesis-related genes. Moreover, *in vitro* studies further confirmed the osteogenic role of Sema3A in the T2DM model. It is notable that the Sema3A group had relatively few new blood vessels at 4 weeks after surgery compared with the control group. This may be related to the fact that the receptor NRP-1 is shared by Sema3A and vascular endothelial growth factor (VEGF). Many studies on tumors have shown that Sema3A competes with VEGF for the receptor NRP-1, which inhibits VEGF-mediated angiogenesis, thereby further inhibiting the growth, invasion, and metastasis of tumor [40]. However, 8 weeks after surgery, the difference in the number of blood vessels between the two groups was not obvious. The possible mechanism may be that Sema3A injected locally on days 1, 4, and 7 may decrease as time flows and the autocrine or paracrine VEGF of ASCs may increase at the later stage.

The present study demonstrated that ASC sheets and the Bio-Oss® bone granule complex combined with a local injection of Sema3A can greatly promote bone healing under T2DM conditions.

## 5. Conclusion

Our study has identified that rat ASC sheets have stronger osteogenic ability than ASCs *in vitro*. ASC sheets combined with Bio-Oss® bone granules promoted bone formation in T2DM rats. In addition, Sema3A promoted the osteogenic differentiation of ASC sheets *in vitro* and local injection of Sema3A promoted T2DM rats' calvarial bone regeneration based on ASC sheets and Bio-Oss® bone granule complex treatment. Our study provided a novel strategy with high efficiency and convenience to perform bone tissue engineering only by mixing cell sheets with scaffold granules. Moreover, bone tissue engineering based on ASC sheets combined with a local injection of Sema3A provides a promising strategy to repair bone defects in T2DM patients.

## Figures and Tables

**Table 1:** Primers used for real-time quantitative polymerase chain reaction.

Gene	Forward primer sequence (5′-3′)	Reverse primer sequence (5′-3′)
GAPDH	CAAGTTCAACGGCACAGTCA	CCATTTGATGTTAGCGGGAT
ALP	ATGGCTCACCTGCTTCACG	TCAGAACAGGGTGCGTAGG
BMP2	GAGGAGAAGCCAGGTGTCT	GTCCACATACAAAGGGTGC
OCN	CCACCCGGGAGCAGTGT	GAGCTGCTGTGACATCCATACTTG
OPG	ACAATGAACAAGTGGCTGTGCTG	CGGTTTCTGGGTCATAATGCAAG
RUNX-2	GCACCCAGCCCATAATAGA	TTGGAGCAAGGAGAACCC

## Data Availability

The data used to support the findings of this study are available from the corresponding author upon request.

## Abbreviations

ASCs: Adipose-derived stem cells
ASC sheets: Adipose-derived stem cell sheets
T2DM: Type 2 diabetes mellitus
BMSCs: Bone marrow mesenchymal stem cells
PDLSCs: Periodontal ligament stem cells
PBS: Phosphate-buffered saline
*α*-MEM: *α*-Minimum essential media
ALP: Alkaline phosphatase
PE: Phycoerythrin
FITC: Fluorescein isothiocyanate
OPG: Osteoprotegerin
BMP2: Bone morphogenetic protein 2
OCN: Osteocalcin
RUNX-2: Runt-related transcription factor 2
STZ: Streptozotocin
CSD: Critical-sized calvarial defect
ECM: Extracellular matrix
HA: Hydroxyapatite
Micro-CT: Microcomputed tomography
ROI: Region of interest
Hu: Hounsfield units
BV: Bone volume
TV: Total volume
Tb.Th: Trabecular thickness
Tb.N: Trabecular number
Tb.Sp: Trabecular spacing
Sema3A: Semaphorin 3A
VEGF: Vascular endothelial growth factor.
